# Mechanistic Design of Cell-Penetrating Disruptors for a Phospho-Dependent Interaction

**DOI:** 10.21203/rs.3.rs-6862805/v1

**Published:** 2025-06-17

**Authors:** Vanda Gunning, Matthew Batchelor, Krista K. Alexander, Martin Walko, Selena G. Burgess, Stephen J. Royle, Eileen J. Kennedy, Richard Bayliss

**Affiliations:** 1. Astbury Centre for Structural Molecular Biology, University of Leeds, Leeds, United Kingdom; 2. School of Molecular and Cellular Biology, Faculty of Biological Sciences, University of Leeds, Leeds, United Kingdom; 3. Division of Chemical Biology and Medicinal Chemistry, Eshelman School of Pharmacy, University of North Carolina at Chapel Hill, Chapel Hill, United States; 4. School of Chemistry, Faculty of Engineering and Physical Sciences, University of Leeds, Leeds, United Kingdom; 5. Centre for Mechanochemical Cell Biology, Warwick Medical School, University of Warwick, Coventry, United Kingdom

## Abstract

The complex formed by transforming acidic coiled coil 3 (TACC3) and clathrin heavy chain (CHC) enhances mitotic spindle stability and strength by cross-linking microtubules. The interaction is dependent on phosphorylation of TACC3 at S558 by Aurora-A. Previously, we elucidated the structural basis of the TACC3/CHC interaction, which is driven by hydrophobic residues on both proteins and the formation of an α-helix in TACC3 that docks into the helical repeats of CHC. Here we find that this phosphorylation event plays an unusual role in the protein–protein interaction; rather than direct bond formation, the phosphorylated residue acts by overcoming an inherent electrostatic repulsion between K507 of CHC and basic residues in TACC3. Leveraging this insight, we optimized the sequence using peptide arrays to develop a hydrocarbon-stapled peptide (SP TACC3) that binds CHC with over a hundred-fold higher affinity than the parental TACC3 peptide, effectively disrupting the native interaction. The crystal structure of the SP TACC3/CHC complex reveals the basis for the enhanced interaction and highlights the contribution of additional polar and hydrophobic interactions. SP TACC3 efficiently penetrates cells and displaces TACC3 from the mitotic spindle, causing a delay in mitotic progression in two out of three cancer cell lines. This work showcases the novel application of hydrocarbon-stapled peptides to disrupt the TACC3/CHC protein–protein interaction in a cellular context, highlighting the potential of targeting this interface for future cancer therapies.

## Introduction

Phosphorylation is a key protein modification that enables the dynamic regulation of activity, cellular localisation and stability of the majority of mammalian proteins. The rapid and reversible addition or removal of a dianionic phosphate group induces changes of the protein structure and dynamics^1–4^. Phosphorylation regulates various cellular processes including cell differentiation, development and the cell cycle. It is also crucial for protein–protein interactions (PPIs) within cell signalling pathways and can control disorder-to-order transitions upon binding to a protein partner, providing high specificity in often low affinity interactions^5–9^. Phospho-binding proteins recognise phosphorylation through specific domains, including SH2 domains^10–12^, PTB (phosphotyrosine binding) domains^13,14^, 14-3-3 proteins^15^ and WW domains^16^. Most phosphorylation sites are located in intrinsically disordered proteins (IDPs) or disordered protein regions^8,17,18^. The unstructured nature of these proteins or regions facilitates these post-translational modifications, and subsequently, the functions of IDPs are regulated by the phosphorylation event. The effects of the modifications involve changes in association state, activation or inhibition of a protein and transition between disorder and order^3,19–21^.

Transforming acidic coiled-coil 3 (TACC3), belonging to the TACC family of centrosome proteins, is a largely disordered protein with a highly conserved C-terminal coiled-coil domain^22^. It is one of the essential regulators of centrosome integrity as well as mitotic progression, promoting microtubule stability during mitotic spindle assembly in mammals^23–25^. In human cells TACC3 is phosphorylated on residue S558 by the mitotic kinase Aurora-A, and consequently binds to the heavy chain of clathrin (CHC), which partially localises from the cytoplasm to growing spindles during mitosis^26,27^. The complex of phosphorylated TACC3 (pTACC3) with clathrin is proposed to form a microtubule binding surface consisting of the coiled-coil region of TACC3 and the β-propeller domain of CHC. This complex localises along the spindle, forming bridges between parallel microtubules of the kinetochore fibres (k-fibres), physically crosslinking k-fibres and contributing to spindle stability and strength^23,26–30^. Unphosphorylated TACC3 is targeted to the plus-ends of microtubules by ch-TOG independently of Aurora-A and CHC^31,32^.

The structural basis of the pTACC3/CHC interaction, involving hydrophobic interactions and a conformational transition of pTACC3, was established in previously published work^26^. The CHC-interaction domain of TACC3 (aa 549–570, CID), which includes the S558 site, acquires an α-helical conformation upon binding to CHC; Aurora-A must phosphorylate S558 to initiate the interaction. The core of the interface is hydrophobic, involving the side chains of TACC3 residues L559, Y560, F563 and P565, which interact with residues L476, L480, R481, L503, Y504, and K507 of the CHC on the interaction surface formed by α10 and α12 helices^26^. Additionally, the TACC3 di-leucine motif L566/L567, whose interacting partners were not identified, is essential for interaction with CHC during spindle localisation^33^. TACC3 is overexpressed in a variety of cancers and therefore represents a therapeutic target for the treatment of the respective malignancies^25,34–38^. Recent studies show that in order to disrupt microtubule stability, inhibitors need to target the TACC3/CHC interaction or the interface between the TACC3/chTOG/CHC complex and microtubules^39^.

Although protein–protein interaction interfaces often lack druggable pockets, inhibitory cell permeable peptides, which have emerged as a promising therapeutic strategy, show a propensity to bind shallow protein surfaces^40–42^. In particular, hydrocarbon stapled α-helical peptides may increase α-helical content and enhance target binding and cell penetration in combination with increased protease resistance^42–45^. To design a peptide inhibitor of PPIs a detailed mechanism of the protein complex formation is required. In many cases the regulation of interaction is difficult to fully clarify, preventing the understanding of IDP function and the search for druggable IDP targets^46^.

Utilising structural data and biophysical analyses, we investigated the role of phosphorylation in the interaction between TACC3 and CHC, particularly in the context of the conformational transition of TACC3 CID and the absence of a conventional phospho-reader domain in CHC. We exploited the high specificity of TACC3 CID binding to an extended surface of the CHC ankle region and developed a stapled, cell permeable peptidomimetic inhibitor that disrupts this interaction within the cellular environment. The development of this novel probe disrupting the TACC3/CHC interface in a cellular context highlights the significance of this interface as a potential cancer therapeutic target.

## Methods

### DNA manipulation

CHC constructs for binding studies were produced during previous work by cloning the respective CHC coding sequence of Q00610 (CLH1_HUMAN) aa 1–574 and 358–574, respectively) into a modified pGEX vector containing a TEV-cleavable GST tag^26^. Point mutations K506A and K507A were introduced by site-directed mutagenesis. GB1-TACC3 is the small protein GB1^47^ fused to a short, disordered peptide sequence from human TACC3 (Q9Y6A5, aa 545–573). This hybrid sequence was cloned into a pETM6T1 vector which contains a TEV protease-cleavable N-terminal His-NusA tag.

### Protein expression and purification

GST-tagged CHC proteins and a biotinylated version of CHC aa 358–574 were expressed and purified using standard procedures as described in previous work^48^. GB1-TACC3 was expressed in BL21 (DE3) RIL *Escherichia coli* cells. 50 μg/mL kanamycin was used to select and maintain the pET vector; 35 μg/mL chloramphenicol was used for routine maintenance of the RIL vector. A single colony was grown overnight in 50 mL of LB at 37 °C. This starter culture was used to seed 1 L of LB media. Cells were grown to an OD of ~0.6, then pelleted at 2500*g*, resuspended in PBS buffer to remove residual LB, pelleted again and resuspended in 250 mL of minimal media. Minimal media for ^15^N/^13^C expression of GB1-TACC3 consisted of 2 g/L of ^15^NH_4_Cl and 4 g/L of ^13^C D-glucose in 50 mM Na_2_HPO_4_, 25 mM KH_2_PO_4_, 20 mM NaCl, 2 mM MgSO_4_, 0.2 mM CaCl_2_, 0.01 mM FeSO_4_, supplemented with micronutrients and vitamins (BME vitamins, Sigma-Aldrich). For ^15^N-only expression, standard glucose was used. After transferral to an autoclaved 2.5 L flask, cells were incubated at 20 °C for a further 2 h at 200 rpm and then overnight expression was induced with 1 mM IPTG at 20 °C. Cells were harvested by centrifugation and pellets stored at −80 °C. Cell pellets were thawed and resuspended in TBS (20 mM tris, 150 mM NaCl, 2 mM β-mercaptoethanol, pH 8) supplemented with a c0mpleteTM Mini EDTA-free Protease Inhibitor Cocktail tablet (Roche). Cells were lysed by sonication and then clarified by centrifugation at 40,000*g* for 45 min. Protein was purified from lysate using Ni-affinity chromatography with a HisTrap column; eluting from the column using a 0–500 mM gradient of imidazole. TEV protease was added to NusA-GB1-TACC3 containing fractions, and the solution was dialysed in TBS buffer at 4 °C overnight. The solution was subjected to Ni-affinity subtraction step to remove the cleaved His-NusA tag and His-tagged TEV protease. GB1-TACC3 protein was further purified and buffer-exchanged using size-exclusion chromatography (SEC) with a Superdex 16/600 S75 column (GE Healthcare). The final size exclusion buffer contained 20 mM (K/H)_3_PO_4_, 150 mM NaCl, pH 6.5. GB1-TACC3 was concentrated, flash frozen in liquid nitrogen and stored at −80 °C. Aurora-A (aa 122–403) and TPX2 (aa 1–43) were prepared via previously described methods^31^.

### Stapled peptide synthesis

The stapled peptide synthesis was carried out using Fmoc solid phase peptide synthesis (SPPS). Peptide sequences were generated on Rink Amide MBHA resin (Novabiochem) using N-α-Fmoc amino acids (Novabiochem). At the N-terminus, peptides were labelled with FAM (5[6]-carboxyfluorescein) (Sigma-Aldrich). Prior to each coupling reacting, a deprotection step containing 25% piperidine (Fisher Scientific) in N-methyl pyrrolidinone (NMP) (Fisher Scientific) was applied to the sequence for 25 min followed by three 30 s washes in NMP. To couple each amino acid to the sequence, 10 equivalents of amino acid, 9.9 equivalents of O-(1H-6-chlorobenzotriazole-1-yl)-1,1,3,3-tetramethyluronium hexafluorophosphate (HCTU) (Oakwood-Chemical), and 20 equivalents of N,N-diisopropyl ethylamine (DIEA) (Fisher Scientific) were applied for 45 min. To couple the olefinic (S)-N-Fmoc-2-(4-pentenyl) alanine (S5) (Sigma-Aldrich) amino acid to the sequence, 4 equivalents of the amino acid were combined with 3.9 equivalents HCTU and 20 equivalents of DIEA. Prior to labelling the sequence with FAM, ring-closing metathesis was performed using 0.4 equivalents of Grubb’s I catalyst (Sigma-Aldrich) dissolved in 1,2-dichloroethane (DCE) (Fisher Scientific) for 1 h. Following coupling, sequences were labelled with FAM as follows: 2 equivalents of FAM (5[6]-carboxyfluorescein) were dissolved in dimethylformamide (DMF) (Fisher Scientific) and combined with 1.8 equivalents HCTU and 4.6 equivalents DIEA. The reactions were carried out overnight. After adding all amino acids and labels to the sequences, the peptides were cleaved from the resin using 95% trifluoroacetic acid (TFA) (v/v) (Fisher Scientific), 2.5% triisopropylsilane (TIPS) (v/v) (Fisher Scientific), and 2.5% water (v/v). This solution was agitated for 4–5 h at room temperature, then filtered into ice-cold methyl-tert-butyl ether (MTBE) (Fisher Scientific) and centrifuged at 2,800 rpm for 30 min. Following centrifugation, the supernatant was removed, and the pellet was air dried. The peptide pellets were subsequently dissolved in methanol (Fisher) and filtered through a 0.45 μm syringe filter prior to purification.

### Identification, purification and quantification of peptides

Peptides were purified by reversed-phase high-performance liquid chromatography (RP-HPLC, Agilent 1250) using a semi-prep Zorbax SB-C18 column with a flow rate of 4 mL/min. A 10–100% gradient of acetonitrile and 0.1% TFA in water was used in the mobile phase. Peptide products were identified by electrospray-ionization mass spectrometry (ESI-MS) (Agilent 6120 Single Quadrupole) at a flow rate of 0.5 mL/min. FAM labelled peptides were quantified on a microplate reader (BioTek) at 495 nm using 10 mM Tris buffer (pH 8.0) and an extinction coefficient of 69,000 M^−1^ cm^−1^.

### Fluorescence polarisation direct binding assay

Binding affinity of CHC and TACC3 peptides was calculated based on fluorescence polarization assays performed as reported previously^26^, using linear and stapled FAM-TACC3 peptides corresponding to aa 549–570 of human TACC3 protein. Linear peptides were purchased from PPR (Fareham, UK); Alta Bioscience (Redditch, UK); Biomatik Corporation (Ontario, Canada). Stock solutions of peptides were 10 mM in DMSO. Binding assays were performed in triplicate in the binding buffer 20 mM HEPES pH 7.5, 150 mM NaCl, 5 mM DTT, 0.05% Tween-20. Data analysis was performed using a one-site specific binding model in Prism (GraphPad software, v10.1.0).

### Peptide arrays

Peptide arrays were synthesized on 10×15 cm cellulose membrane made of 6-aminohexanoic acid modified Whatman 540 filter paper, using MultiPep 2 peptide synthesizer (CEM Corporation). First, Fmoc-8-amino-3,6-dioxaoctanoic acid spacer was coupled to the membrane to provide a distance between the solid support and the peptide. Peptides were then assembled using standard Fmoc-based solid phase synthesis with double deprotection step (2 × 15 min) using 20% piperidine in DMF, double couplings (2 × 30 min) with N,N’- diisopropylcarbodiimide (DIC) and OXYMA as coupling reagents and 10% acetic anhydride in DMF as caping reagent (15 min). Final deprotection was achieved by incubating the membrane in the mixture of 92.5% TFA, 2.5% water, 2.5% TIPS and 2.5% 3,6-dioxa-1,8-octanedithiol (DODT) for 3 h, followed by three dichloromethane, three DMF, three ethanol washes and air-drying.

The peptides on the membrane were rehydrated in ethanol for 5 min followed by 5 min wash in TBST (50 mM Tris pH 7.5, 150 mM NaCl, 0.05% Tween-20). The membrane was blocked by 1% bovine serum albumin (BSA) in TBST with agitation for 1 h and washed for 5 min in the binding buffer (20 mM HEPES pH 7.5, 150 mM NaCl, 5 mM DTT, 0.05% Tween-20). The target protein biotinylated clathrin 358–574 was diluted into the binding buffer to a concentration of 1 μM and incubated with the membrane for 1 h at room temperature with agitation. After washing the membrane 3 × 5 min with TBST, Anti-biotin HRP-linked Antibody (7075S, Cell Signalling) was added to the membrane (1:1500 dilution) and incubated for 1 h with agitation. After another 3 washes in TBST the spots were visualised by ECL Western Blotting Substrate (Fisher Scientific) using an iBright instrument (Fisher Scientific). After subtracting the background signal, intensity data were normalised and expressed as a percentage compared to the intensity of the strongest binding peptide.

### Competitive pull-down assays

For *in vitro* peptide pull-down assays, 50 μL of Strep-Tactin XT Sepharose beads (IBA) were incubated in 500 μL of pull-down buffer (20 mM HEPES pH 7.5, 150 mM NaCl, 5 mM DTT, 0.05% Tween-20) with 100 μM N-biotinyl-WT pTACC3 peptide (Biomatik Corporation, Ontario, CA) for 1 h at room temperature and then washed three times. The beads were then incubated with 500 μL buffer containing 0.6 mg/mL CHC ankle region protein (aa 358–574) and competing FAM-labelled peptides (50 nM) (PPR, Fareham) for 1 h at room temperature. After the incubation the beads were washed 3 times with 900 μL pull-down buffer and finally, the bound proteins were eluted by boiling with 30 μL pull-down buffer + 10 μL 4x LDS-loading buffer (Invitrogen). The eluted proteins were analysed by SDS/PAGE. Before staining, the blots were visualised on an iBright imaging system (Fisher Scientific) to quantify the FAM-labelled peptides in the samples, other interacting proteins were quantified after Coomassie staining using the same imaging system (iBright, Fisher Scientific).

### SPR

The SPR assays were performed using a Biacore 1K+ instrument (Cytiva Life Sciences). The biotinylated Avi-tagged ankle region of clathrin (aa 358–574) was immobilised on the surface of a Streptavidin SA chip (Cytiva Life Sciences) to the density of 150 RU. The increasing concentrations of peptide analytes were diluted in the running buffer (20 mM HEPES pH 7.5, 150 mM NaCl, 5 mM DTT, 0.05% Tween-20) and injected at a flow rate of 30 μL/min). Serial dilutions of the peptides were made spanning concentrations from 0.6 nM to 0.7 μM for Opt SP TACC3, and 48 nM to 50 μM for WT TACC3 and SP4E. Each concentration was injected at 30 μL/min for 60 s. The dissociation step was 360 s without regeneration of the chip surfaces, as clathrin was found to be affected by regeneration solutions. The Biacore evaluation software was used to calculate equilibrium K_D_ values as well as *k*_a_ and *k*_d_ constants using 1:1 steady-state affinity model implemented into the BIAevaluation 3.0 software.

### CD

CD spectra were recorded using an APP Chirascan CD spectropolarimeter and 1 mm pathlength quartz cuvettes. N-terminally acetylated and C-terminally amidated TACC3 and pTACC3 peptides (Q9Y6A5, aa 549–570) were purchased from PPR (Fareham, UK). Some additional, equivalent samples of unlabelled TACC3 and pTACC3 were synthesised and purified in house by Dr Robert Dawber following a previously reported protocol^49^. Solid peptide samples were dissolved in CD buffer (1 mM sodium borate, 1 mM sodium citrate, 1 mM sodium phosphate, 10 mM NaCl, pH 7.2) to 1 mM stock levels and concentrations recorded using A_280_ measurements. For CD measurements, these stock solutions were further diluted in CD buffer to 50–100 μM. This buffer alone has low absorbance in the region of wavelengths (190–260 nm) used for CD measurements. For all other peptides, sample concentrations of 5 μM were achieved by diluting 10 mM DMSO stock solutions with CD buffer. Some disruption to data collection at wavelengths <200 nm was observed due to the DMSO present in solution. Spectra were recorded at 5 °C over wavelengths ranging from 260 to 180 nm, with data collected every 1 nm (1 nm s^−1^), and each spectrum was recorded in duplicate. A background spectrum for CD buffer alone or CD buffer with 0.2% DMSO was recorded for each data collection session. MREs values were calculated as described previously^49^.

### NMR

Spectra for unlabelled TACC3 peptides were recorded on a 950-MHz Bruker Ascend Aeon spectrometer equipped with a 5 mm TXO cryoprobe (additional experiments were carried out using a 600-MHz Oxford Instruments spectrometer equipped with a 5 mm Bruker QCI-P cryoprobe and a Bruker Avance III HD console). Natural abundance ^1^H–^15^N HMQC and ^1^H–^13^C HSQC (constant-time) spectra were recorded for ~0.8 mM samples at 10 °C in Tris buffer (20 mM Tris, 100 mM NaCl, 2 mM KCl, pH 7.4, 5% D_2_O). Assignments were made, as far as possible, using ^1^H–^1^H TOCSY and ^1^H–^1^H NOESY spectra. Data acquisition was achieved throughout using Topspin, spectra were generated with NMRPipe/NMRDraw (10.1007/BF00197809), and CCPNmr Analysis v2.5 (10.1002/prot.20449) was used for peak assignment and further analysis. *In situ* phosphorylation of a 240 μM sample of TACC3 CID in CD buffer containing 100 mM NaCl, 3 mM MgCl_2_, 1 mM ATP, 1 mM DTT and 5% D_2_O was achieved by addition of 1 μM Aurora-A (aa 122–403) at 22 °C. Spectra were equivalent to synthetically generated pTACC3 CID.

Spectra for assignment of GB1-TACC3 were recorded on the 600-MHz spectrometer. ^1^H–^15^N HSQC spectra were recorded using a 0.8 mM sample of ^15^N/^13^C-labelled GB1-TACC3 (20 mM (K/H)_3_PO_4_, 150 mM NaCl, 5% D_2_O, pH 6.5) at 10 °C. HNCO, HNcaCO, HNCA, HNcoCA, HNcaCB and HNcocaCB (HNC) triple-resonance assignment spectra and a HBHAcoNH, ^1^H–^15^N-NOESY-HSQC and ^1^H–^13^C HSQC spectrum were recorded. Full *ab initio* assignment of the ^1^H–^15^N HSQC spectrum was achieved; the GB1 assignment subsequently being cross-referenced with known assignments. Peak positions were manually tracked to transfer assignments to HSQC spectra recorded at higher temperatures.

Rapid i*n situ* phosphorylation (~minutes) of 140 μM ^15^N(^13^C)-labelled GB1-TACC3 was achieved through addition of MgCl_2_ (3 mM), ATP (2 mM), Aurora-A (122–403) (2 μM) and TPX2_1–43_ (2 μM). ^1^H–^15^N HSQC spectra were recorded on a 750-MHz Oxford Instruments magnet equipped with a Bruker Avance console and TCI cryoprobe. Phosphorylation without TPX2, as determined via emergence of the pS558 peak and alterations in peak position for neighbouring residues, was very slow in this buffer (~hours/days). Rapid phosphorylation without TPX2 was achieved in ‘kinase buffer’ (20 mM Tris, 25 mM NaCl, 1 mM MgCl_2_, 0.01% Tween-20, pH 7.5, 5% D_2_O) but at the expense of spectral quality. Peak positions were not significantly altered between buffers. CHC was titrated into ~140 μM GB1-TACC3 or GB1-pTACC3 samples until significant peak intensity losses in ^1^H–^15^N HSQC spectra were observed (1:5 CHC:TACC3 molar ratio).

### Crystallization

CHC (aa 1–574) was concentrated to 20 mg/mL (300 μM), mixed with Opt SP TACC3 peptide (400 μM) and incubated at RT for 15 min prior to crystallization trials. The complex was screened against a range of commercial crystallization matrices. Drops were laid down at a 1:1 and 1.5:1 ratio of complex:precipitant in MRC sitting drop plates using a Mosquito LCP crystallization robot (SPT labtech) and incubated at 18 °C. Crystals were produced using JBScreen JCSG++ (Jena Bioscience) condition B12 (20% w/v polyethylene glycol 3350, 200 mM tri-potassium citrate; pH 8.3) after 2–7 days. Diffraction-quality crystals were obtained after 7 days using the hanging drop vapour diffusion method. The complex was crystallized by mixing 1.5 μL protein with 1 μL reservoir solution (20% w/v polyethylene glycol 3350, 200 mM tri-potassium citrate; pH 8.3). Crystals were flash-frozen in liquid nitrogen with the addition of 20% ethylene glycol or 20% glycerol to the mother liquor to act as a cryoprotectant. Diffraction data were collected from a single crystal at Diamond Light Source (Oxford, UK) on beamline i04. Data was reduced using the autoprocessing pipeline (STARANISO), and the structure determined using PHASER with the search model 5ODS^50,51^. The model was rebuilt using Coot and refined using REFMAC5^52,53^. The molecular coordinates and data have been deposited in the Protein Data Bank, with accession code 9R9B.

### Cell culture

Cell lines used in the study: HeLa (ATCC), MDA MB 468 (ATCC) and MDA MB 231 cells (ATCC). Cell lines were checked for mycoplasma contamination. HeLa and MDA MB 231 cells were maintained in Dulbecco’s modified Eagle medium (DMEM) (Gibco) supplemented with 10% heat-inactivated foetal bovine serum (FBS) (Gibco). MBA MB 468 were grown in Roswell Park Memorial Institute (RPMI) medium with 10% FBS at 37 °C in a 5% CO_2_ atmosphere.

### Flow cytometry

HeLa, MDA-MB-468 and MDA-MB-231 cells were plated in 12-well plates at the density of 1.5×10^5^ and allowed to adhere for 24 h in complete growth medium (DMEM and RPMI, respectively, supplemented with L-glutamine,10% FBS). For peptide uptake measurements the medium was aspirated and cells were treated with fresh pre-warmed complete growth medium supplemented for 60 min with FAM-peptides at concentrations of 1, 2.5, 5 and 10 μM; the final concentration of DMSO was 0.2%. To perform the time course of peptide uptake, cells were treated with fresh growth medium supplemented with 5 μM peptide for 15–360 min. After the incubation, cells were washed three times in ice cold phosphate-buffered saline (PBS) and trypsinised for 5 min to remove surface bound peptides and dissociate cells from the surface. The dissociation reaction was stopped using PBS containing 5% FBS. Cells were harvested by centrifugation (5 min, 150 g) and washed an additional three times in PBS. Cells were then resuspended in 300 μL PBS and subjected to a Cytoflex S cytometer (Beckman Coulter) measurement. Using forward and side scattered light, a gate for intact, non-aggregated cells was defined. The fluorescence at 525 nm of 10,000 events was collected within this cell gate and data were analysed using Cytoflex software (version 10.1). Results are shown as geometric means of fluorescence collected at 525 nm.

### Annexin V assay

HeLa, MDA-MB-468 and MDA-MB-231 cells were plated in 12-well plates at the density of 8×10^4^ and cultured for 24 h in complete growth medium (DMEM and RPMI, respectively, supplemented with L-glutamine,10% FBS). For viability measurements the medium was aspirated and cells were treated with fresh pre-warmed complete growth medium supplemented with 5 and 10 μM peptides for 24 h. After the incubation, the cells from the plate as well as the dead cells in the medium were harvested. The cells were washed twice in PBS with 2% FBS and resuspended in 100 μL Annexin V Binding Buffer with 5 μL Pacific Blue Annexin V and 5 μL propidium iodide (PI) (BioLegend). Cells were incubated for 15 min in the dark and analysed by flow cytometry with a Cytoflex S cytometer (Beckman Coulter). Using forward and side scattered light, a gate for intact, non-aggregated, diploid cells was defined. Finally, samples were analysed on the PI versus Annexin V scatter plot for live, early and late apoptosis. Data were analysed using Cytoflex software (version 10.1).

### Confocal microscopy

HeLa cells grown on glass coverslips in complete growth medium and treated with 2.5, 5 and 10 μM FAM-peptides for 5 h. Cells were washed with PBS and fixed with PTEMF (20 mM PIPES, pH 6.8, 10 mM EGTA, 1 mM MgCl_2_, 0.2% Triton X-100 and 4% paraformaldehyde) for 10 min and permeabilized at room temperature in 0.5% Triton-X100 in PBS for 10 min. Cells were blocked in 3% BSA in PBS for 1 h and incubated overnight with primary antibodies diluted in 3% BSA/PBS buffer followed by 1 h incubation with secondary antibodies. Primary antibodies were as follows: mouse anti-CHC (X22; GTX22731; 1:250), rabbit anti-TACC3 (ab134154, Abcam; 1:500). Secondary antibodies were anti-mouse IgG Atto 594 (1:250, 76085, Merck) and anti-rabbit IgG Atto 647N antibody (1:250, 40839, Merck). Coverslips were mounted with ProLong Gold antifade reagent with DAPI (Invitrogen). Peptides were imaged using their 6-FAM fluorescent tag with a 488 nm argon laser. Imaging was performed on a Zeiss LSM880 upright confocal microscope using a 40x/1.4 NA oil objective. Z-stacks comprising of 9–13 × 1 μm sections were acquired. Images were captured using identical settings. The region of interest (~0.6 μm^2^) was quantified in three different locations over the spindle, cytoplasm and in the non-cell region of background. Images were analysed using Fiji (v.2.9.0).

### Live cell imaging

HeLa, MDA-MB-468 and MDA-MB-231 cells were plated in a 35 mm imaging dish (Ibidi) in complete growth medium. Prior to imaging, the DMEM or RPMI growth medium was replaced with Optimem medium (Gibco) supplemented with 10% FBS. Added to the medium was SiR-tubulin (Spirochrome) at 30 nM concentration, SPY555-DNA at 1:18000 dilution; both live-cell imaging probes were used at lower than recommended concentrations to avoid possible effects on mitosis. 5 μM SP TACC3, 5 μM negative control SP TACC3, or DMSO carrier, respectively, were added at the same time and cells were incubated for 4 h, before placing in the Zeiss Lattice Lightsheet 7 microscope incubator and equilibrated at 37°C, 5% CO2 atmosphere and 80% humidity. The Lattice Lightsheet 7 microscope was fitted with a 10x/0.4NA water immersion illumination lens, a 48x/1.0NA detection lens and a PCO.edge 4.2 sCMOS camera. Each field of view of approximately 500 μm × 297 μm × 33 μm was imaged at 0.2 μm lightsheet interval with the 640 nm channel (15 ms exposure, 15% laser power) and 561 nm channel (15 ms exposure, 10% laser power) over a 10 h period with scans taken every 5 min. To create movies and frame sequences the cell were imaged every 2.5 min for 5 h. Results were processed using Zeiss LLS software and Fiji (v.2.9.0). Prophase was defined from the breakdown of nuclear envelope and centrosome formation until the establishment of the mitotic spindle. Metaphase spanned the period of mitosis with a fully established mitotic spindle until the anaphase onset. Anaphase included the chromatid segregation up to the point of a visible midbody.

### Rosetta modelling

Rosetta^54,55^ was used to generate 50 relaxed structures of the original TACC3/CHC crystal structure model (PDB 5ODS, chains A and E). For compatibility, pS558 in the original structure was replaced with Glu. Using each of these 50 models as a starting structure, 20 new models for mutation to all different residue types at each site in TACC3 as well as a WT repeat were generated using the cartesian-ddG application (https://docs.rosettacommons.org/docs/latest/cartesian-ddG)^56^. The averaged total energy difference for each mutation compared to the averaged WT value for each starting structure was calculated.

### Evolutionary biology

Orthologous sequences of TACC3 were identified via a BLAST search using the UniProt sequence Q9Y6A5 TACC3_Human as a template and in UniProt by constraining the searches to a specific taxonomic group. The multiple sequence alignment of TACC3 522–578 was generated using COBALT (Constraint-based alignment tool for multiple protein sequences)^57^ and the graphical representation of the sequence identity using SnapGene 7.0.3.

## Results

### Phospho-dependent and phospho-independent binding of TACC3 to CHC

Our original analysis showed that pTACC3 adopts a helical conformation in complex with the ankle region of CHC (Fig. S1A)^26^. However, due to the resolution of this crystal structure (3.09 Å), the electron density for some side chains was poorly defined (Fig. S1B). This raised unresolved questions regarding the interactions made by several side chains, including TACC3 pS558 and L556/557 (Fig. S1C). To facilitate interaction studies, we used the minimal ankle region of CHC (CHC^ankle^, aa 358–574) and the TACC3 clathrin-interacting domain (TACC3^CID^, aa 549–570) (Fig. S1C). Fluorescence polarisation (FP) assays were used to assess the binding of a series of TACC3^CID^ variant peptides to CHC^ankle^ protein. These studies confirmed the key role of specific hydrophobic side chains on the binding interface, namely L559, Y560, F563 and L566, as critical for binding (Fig. S1D, E).

**Fig. 1:**
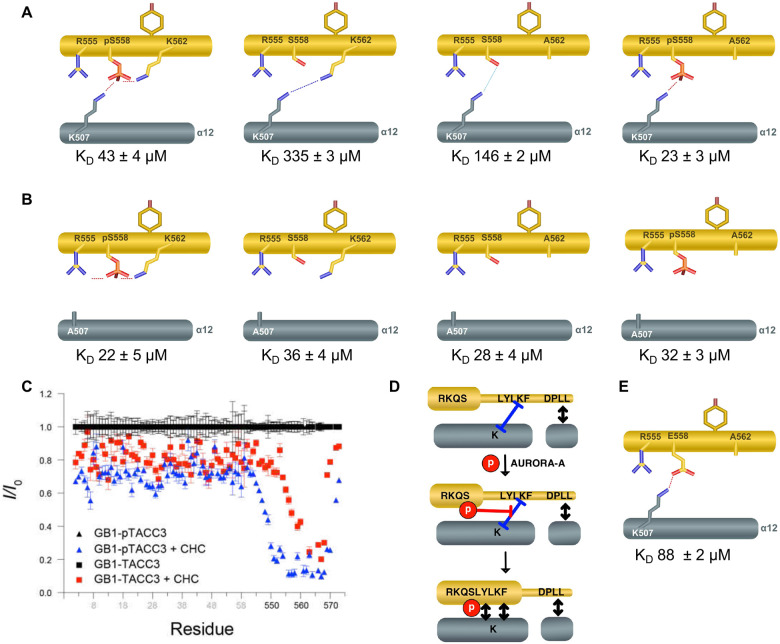
TACC3 phosphorylation enhances binding affinity for CHC by overcoming electrostatic repulsion. **A** Schematic illustrations of CHC (grey) and TACC3 (yellow) interactions and the binding affinities determined using fluorescence polarization assays (CHC^ankle^, TACC3^CID^). **B** As A, but with the K507A variant of CHC. **C** Comparison of the effect on ^1^H–^15^N HSQC peak intensity upon addition of CHC to GB1-TACC3 (red) or GB1-pTACC3 (blue). In both cases, the final concentrations of TACC3 and CHC were ~130 μM and ~25 μM. **D** Schematic illustration of CHC (dark grey) interactions with TACC3 (yellow) in unphosphorylated (upper image) and phosphorylated (lower image) states. **E** Schematic illustration and the binding affinity of the interaction of CHC^ankle^ (grey) and phosphomimetic S558E TACC3^CID^ (yellow) variant with K562 substitution.

We also confirmed that phosphorylation of TACC3 CID S558 increases the binding affinity for CHC^ankle^ approximately 8-fold compared to non-phosphorylated peptide (Fig. 1A, K_D_ 43 μM and 335 μM, respectively). Phosphomimetic glutamic acid is not a functional substitution of pS558 (K_D_ 314 μM) and neither is phosphothreonine (K_D_ 175 μM), consistent with a destabilising effect on helix formation (Fig. S1D, E)^49^. We therefore investigated the contribution of the proposed intramolecular salt-bridge to the helical conformation involving residues R555 ^pTACC3^, K562 ^pTACC3^ and pS558 ^pTACC3 26^. Contrary to our previous studies with a longer CHC protein (aa 1–574), the substitution R555A did not reduce binding of pTACC3 CID to CHC, while K562A further improved binding affinity (R555A^pTACC3^ K_D_ 52 μM, K562A ^pTACC3^ K_D_ 23 μM, R555A K562A ^pTACC3^ K_D_ 26 μM, compared to WT pTACC3 K_D_ 43 μM) (Fig. S1E). These data indicate that the proposed intramolecular salt-bridge in TACC3 appears to not be critical for interactions with CHC^ankle^, as we originally proposed. We therefore sought to revise our model, beginning with a detailed analysis of potential interactions of pS558 with CHC.

In the original CHC (1–574)/pTACC3 crystal structure, pS558^TACC3^ is close to residues K506 and K507 on the neighbouring CHC helix α12 (Fig. S1C)^26^. However, these and other side chains surrounding pS558 had poor electron density suggestive of a dynamic interaction between the proteins or resulting from low-resolution data (Fig. S1B). We investigated the role of K506^CHC^ and K507^CHC^ in the interaction by substituting Lys with Ala and repeated the FP assays with the variant TACC3 CID peptides (Fig. 1A, Fig. S1D, E). The substitution K506A-CHC^ankle^ did not notably impact protein binding, consistent with its side chain orientation being on the side of CHC helix α12 facing away from TACC3 (Fig. S1E). In contrast, the affinity of the K507A-CHC^ankle^ variant for the phosphorylated TACC3 CID peptides was enhanced ~2-fold, indicating that the role of K507^CHC^ is not primarily in recognition of the phosphate group on TACC3 CID (Fig. 1B, S1E). Strikingly, K507A-CHC^ankle^ protein bound unphosphorylated WT TACC3 CID with a K_D_ of 36 μM, almost 10-fold stronger than the WT CHC/TACC3 interaction (K_D_ 335 μM), and similar to the WT CHC/pTACC3 interaction (K_D_ 43 μM) (Fig. 1A, B). This indicates a central role of K507^CHC^ in the selective binding of phosphorylated TACC3. We noted that K562A variants of TACC3^CID^ had enhanced binding to WT CHC^ankle^, but not to the K507A-CHC^ankle^ variant (Fig. 1A, B).

This finding led to the hypothesis that the hydrophobic core at the interaction interface of unphosphorylated TACC3 allows for a weak interaction of the proteins (K_D_ 335 μM) but cannot counteract the repulsive forces of the charged lysine residues (K507^CHC^ and K562^TACC3^) (Fig. 1A, B). The larger negatively charged phosphate, but not a single charge of glutamic or aspartic acid, appears to be needed to neutralise the positive charges of the two Lys side chains, leading to prevention of the electrostatic repulsion and enhanced binding (Fig. 1B). To probe this model, we added CHC^ankle^ to samples of phosphorylated and unphosphorylated ^15^N-labelled GB1-TACC3 CID and collected ^1^H–^15^N HSQC data. The interaction-associated reduction in peak intensities of GB1-TACC3^CID^ are not only more significant for phosphorylated TACC3^CID^ compared to unphosphorylated TACC3^CID^, but the interface extends further towards the N-terminus (Fig. S2). Thus, phosphorylation-independent binding of CHC^ankle^ to TACC3^CID^ is centred on the DPLL-motif^33^, and the phosphorylation-dependent component is in the helical region of TACC3 in the vicinity of the phospho-site.

To probe this mechanism further, we revisited the effect of S558 phosphorylation on the conformation of TACC3. Measurements of helicity for TACC3 CID and pTACC3 CID were carried out using circular dichroism (CD) and NMR spectroscopy (Fig. S2). TACC3 CID peptides (aa 549–570) exhibited very low helicity and there was no increase in overall helicity on phosphorylation (Fig. S2A). However, there was a change in the distribution of helical propensity around the phospho-site, as shown by analysis of Hα chemical shifts in the TACC3 CID peptide (Fig. S2B, C), and Cα secondary shifts in a larger fragment of TACC3 (GB1 fused with aa 546–573 TACC3) (Fig. S2D-F). In both sets of samples, residues N-terminal to the phosphosite showed a decrease in helical propensity on phosphorylation, and residues 559–561, C-terminal to the phosphosite, increased helical propensity. These observations are consistent with the helical conformation of this region of TACC3 in the original CHC (1–574)/pTACC3 crystal structure. Our revised model suggests phosphorylation of S558 expands a small interface involving the C-terminal residues of TACC3^CID^ into a helical region immediately C-terminal to the phosphosite, by overcoming the electrostatic repulsion between basic residues on CHC and TACC3 (Fig. 1D).

This revised model of the interaction helped us to move forward in our inhibitor design, recognising the opportunity to substitute sub-optimal residues and introduce a helical constraint to stabilise the bound form of TACC3, for which phosphorylation is insufficient. The substitution K562A^TACC3^ reduces the repulsion with K507^CHC^ and allows for a stronger interaction of the proteins even when TACC3 is lacking the phosphate, albeit with a lower affinity (K_D_ 146 μM) due to the salt bridge being replaced by a hydrogen bond (Fig. 1A).

### Design of the optimal sequence for a constrained peptide inhibitor

**Fig. 2:**
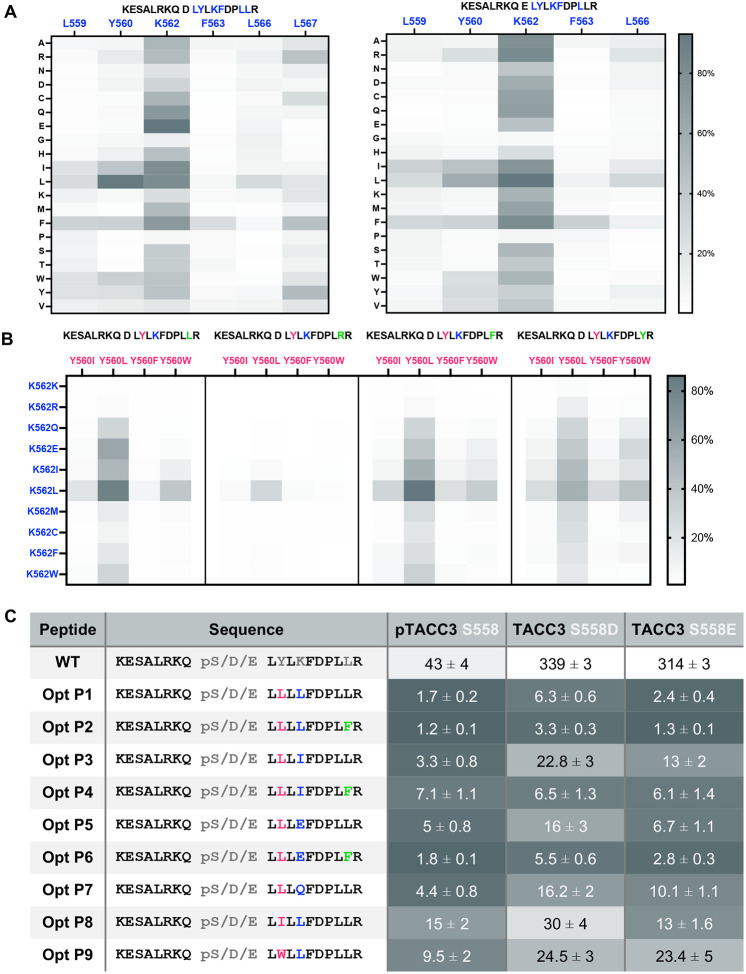
Optimising TACC3 sequence to increase binding affinity to CHC using saturating mutagenesis in peptide array. **A** Heatmap diagram represents relative increase in binding of TACC3 peptide sequences relative to the mean background signal in a color-coded manner from white (no binding) to dark grey (strong binding). Columns represent specific amino acid position in TACC3 sequence (highlighted in blue) substituted by every natural amino acid (rows). **B** Representative heatmap shows relative increase in binding of TACC3 peptides with selected substitutions relative to the background signal - white (no binding) to dark grey (strong binding); each value is an average of two replicates. Columns represent specific amino acid substitution of Y560 in TACC3 S558D sequence (highlighted in magenta), rows represent amino acid substitution of K562 (highlighted in blue), and each block represents a specific L567 substitution (L567, L567R, L567F & L567F) (highlighted in green). **C** Binding affinity of TACC3 peptides with a respective pS558/S558D/S558E substitution (aa 550–568) in the array-optimised sequences to CHC (358–574), measured in direct binding FP assays with FAM labelled peptides. Substituted amino acid are highlighted in magenta, blue and green, respectively; K_D_ values (μM) represent a mean of three experiments ± SD; values are colour-coded from the lowest K_D_ (dark grey) to the highest (white).

Having gained a deeper understanding of how TACC3 protein phosphorylation regulates binding to CHC, we moved forward with designing a constrained peptide inhibitor to disrupt this interaction. Notably, the variant of the unphosphorylated TACC3 peptide, in which the electrostatic repulsion is eliminated through S558E and K562A mutations, had the highest affinity for CHC^ankle^ (K_D_ 88 μM), only a 2-fold reduction in affinity compared to the phosphorylated WT TACC3 sequence (Fig. 1A, E). Therefore, we used phosphomimetic D/E substitutions of pSer at position 558 to optimize a peptide sequence that binds CHC^ankle^ without requiring phosphorylation, as the dianionic, polar phosphate group significantly hinders crossing of the peptide through phospholipid bilayer and impedes cell internalisation^58,59^. The 19-residue core fragment of TACC3 CID essential for the PPI (KESALRKQSLYLKFDPLLR, aa 550–568) was selected for sequence optimisation through two rounds of saturating mutagenesis using peptide arrays. Initially, the key hydrophobic and charged residues were systematically replaced by all twenty natural amino acids (Fig. 2A). This identified residues that form critical interactions with the CHC surface that could not be substituted with an alternative natural amino acid (F563, L566). This experiment also showed sites for potential improvements in binding (Y560, L567). Conversely, the array showed that position 562 is flexible, with most amino acids being functional except for glycine and proline, consistent with the importance of helicity in the bound TACC3 peptide. Flexibility at position 562 was also seen through *in silico* assessment of mutations to the CHC/pTACC3 crystal structure using Rosetta (Fig. S3). The modelling also corroborates the importance of conserving F563, D564 and P565, and is consistent with the peptide array where Y560L was predicted to improve binding.

In the second round of peptide arrays the amino acid substitutions with the highest binding signals were combined into sequences containing double and triple substitutions (Fig. 2B). The Y560L substitution substantially increased binding when combined with K562Q/E/I/L, with either the native L567 or the L567F variant. The combination of Y560I/W with K562L was also effective with the native L567 or L567F variant.

A selection of candidate sequences with enhanced binding to CHC and pS/D/E substitutions at position 558 were synthesised as FAM-conjugates and characterised by FP to establish K_D_ values (Fig. 2C). Binding affinities of the optimised peptides, particularly TACC3 Y560L variants, showed significant improvement of up to forty-fold compared to the WT sequence. Furthermore, the S558E phosphomimetic was a better binder than S558D. Most of the variant peptides that incorporated this Glu substitution had similar binding affinities relative to the pS558 peptide. The peptides Opt P2 and Opt P6 with either pS558 or the S558E substitution had the highest affinities (1–2 μM). These sequence-optimized peptides competed with WT pTACC3 peptides for binding to CHC ankle region in competition coprecipitation experiments (Fig. S4). Notably, the pS558 and S558E variants were more effective than S558D, consistent with their higher binding affinities.

### Constraining the peptide sequence improves binding affinity

**Fig. 3:**
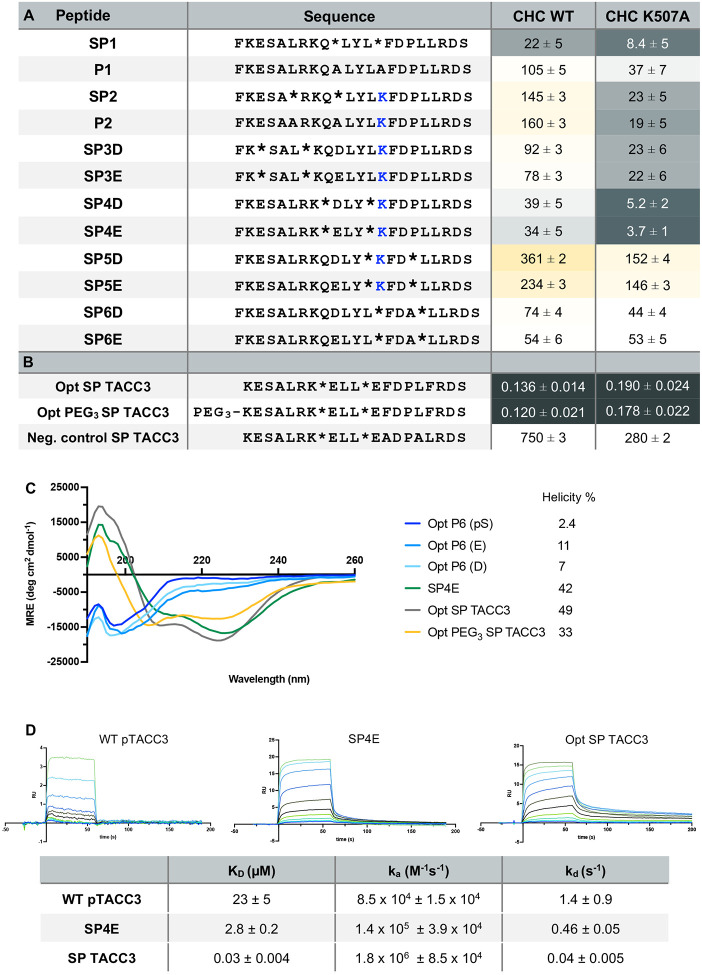
Biophysical characterisation of stapled peptides. **A** Binding affinity of TACC3 stapled peptides originating from WT TACC3 sequences to CHC WT (385–574) and with K507A substitution, measured in direct binding FP assays with FAM labelled peptides. * represents positions where 2-(4′-pentenyl) alanine was inserted into the sequence. Non-stapled peptides bearing modifications were also synthesized as controls (compound P1 and P2). K562^TACC3^ residue is highlighted in blue; K_D_ values (μM) represent mean of three experiments ± SD. **B** Binding affinity of peptides with sequences optimised during peptide array selection. * represents positions where 2-(4′-pentenyl) alanine was inserted into the sequence. K_D_ values (μM) represent mean of three experiments ± SD. **C** Enhanced α-helicity of hydrocarbon stapled peptides compared to unstapled peptides analysed by circular dichroism (CD). CD spectra collected for sequence optimised linear and stapled peptides show the CD signal typical for an **α**-helix in the spectrum of the stapled peptides only. **D** Representative SPR sensorgrams demonstrating dynamics of WT TACC3, SP4E and SP TACC3 peptides interaction with CHC. SPR analyses of peptides binding to CHC show slower association and, in particular, slower dissociation rate of the stapled peptides in comparison to WT pTACC3 sequence; table shows average K_D_, k_a_ and k_d_ ± SD of experiments performed in duplicates.

Stapled peptides have been used to target PPIs mediated by helical protein sequences due to their resistance to proteolytic degradation and improved cell membrane permeability^43,60–65^. The hydrocarbon staple can also contribute to improved binding by pre-organising and constraining the peptide to an α-helix, an approach suitable for a protein acquiring this structure upon binding. To identify the most appropriate position for the staple to improve binding to CHC, a series of TACC3 peptides was synthesised with two monomers of the olefinic amino acid [(*S*)-2-(4-pentenyl)alanine] introduced at several different *i, i +* 4 positions, and the pS558 was replaced with either D or E substitutions. Binding affinities to CHC were determined using the FP assay (Fig. 3A). While constraints at most positions were tolerated, one position had a detrimental effect on the affinity for CHC (SP5 – aa 561, 565), consistent with the Rosetta calculations showing the importance of conserving this residue (Fig. S3). Staple placement near or around the S558 position improved binding to CHC, suggesting that it pre-organised the peptide in a conformation that facilitated improved contact of the hydrophobic residues along the CHC surface. Most of the stapled peptides that retained K562 in their sequences bound with a higher affinity to the K507A variant of CHC, with the exception of the SP5 peptides, consistent with the electrostatic-repulsion model for selective phosphor-TACC3 binding by CHC (Fig. 3A).

In the final stage of the inhibitor design, the staple at the optimal position was introduced to a peptide sequence with optimal substitutions identified from the peptide array (Opt SP TACC3). The chosen sequence (KESALRK*ELL*EFDPLFRDS) for the stapled inhibitor design used the K562E variant, rather than K562L, to overcome the repulsion from K507^CHC^, and to mitigate the risk of poor solubility from having a considerably hydrophobic sequence. As an additional precaution, a second version of the peptide was made with an N-terminal PEG_3_ linker modification (Opt PEG_3_ SP TACC3). The combination of a sequence optimised for stronger binding and a staple positioned on the non-binding face of the helical peptide resulted in inhibitor candidates with nanomolar binding affinities towards CHC (Fig. 3B). Binding was no longer enhanced with the K507A variant of CHC. A negative control stapled peptide based on the optimised SP TACC3 peptide, but with substitutions of key binding interface residues (F563A & L566A) showed only weak binding to CHC as expected (K_D_ ~750 μM).

Circular dichroism (CD) spectroscopy confirmed that the incorporation of the staple resulted in a highly α-helical structure (49% helicity), compared with the unstapled, optimised sequence peptides that were largely disordered (2–11% helicity) (Fig. 3C). Analyses of the binding kinetics using surface plasmon resonance revealed that WT pTACC3 exhibited rapid association and dissociation rates when interacting with clathrin. The stapled peptides, on the other hand, demonstrated a considerably reduced dissociation rate (Fig. 3D).

### The structural basis of enhanced SP TACC3–CHC interaction

**Fig. 4:**
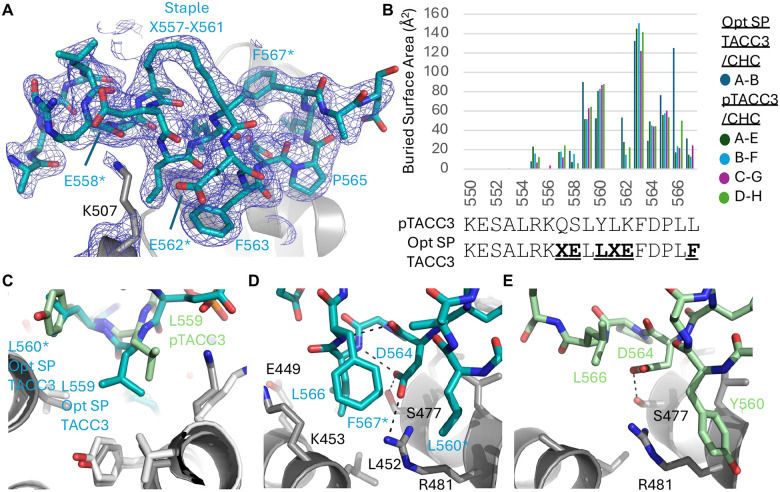
Structural basis of the stapled peptide Opt SP TACC3 bound to CHC. **A** 2Fo-Fc electron density map contoured at 1.0 sigma around Opt SP TACC3. Residues modified in Opt SP TACC3 are indicated with an asterisk. **B** Plot of buried surface area at the CHC interface per-residue along the TACC3 sequence of WT TACC3 and Opt SP TACC3. Residues modified in Opt SP TACC3 are indicated in bold. X represents positions where 2-(4′-pentenyl) alanine was inserted into the sequence. Letters (A-H) specify the chains within their respective asymmetric units of the crystal structures. **C** Superposed structures of Opt SP TACC3/CHC (teal/grey) with pTACC3/CHC (green/white) centred on L559. **D** Interaction surface centred on D564 showing differences in conformation between Opt SP TACC3 and **E** pTACC3 structures.

To understand the molecular basis of its high-affinity interaction with CHC, Opt SP TACC3 peptide was co-crystallized with CHC (1–574) and the X-ray structure determined to 2.15 Å resolution. The electron density map of the peptide in the Opt SP TACC3/CHC complex was greatly improved as compared to the previous WT pTACC3/CHC structure with a limiting resolution of 3.09 Å^26^ (Fig. S6). The hydrocarbon staple is clearly resolved in the peptide electron density map; it makes no contacts with CHC and lies on the opposite face of the helix, as designed (Fig. 4A, S1B). The crystal structure confirms the helical conformation of the peptide and shows the positions of the side chains unambiguously. The helical region of Opt SP TACC3 closely resembles that of pTACC3, despite the lower resolution of the original structure, and the major contributions to buried surface area with CHC are from the same hydrophobic residues (Fig. 4B). Strikingly, L559^Opt SP TACC3^ contributes to a larger buried surface area as compared to the same residue in pTACC3, since it reaches deeper into a hydrophobic pocket formed by L480^CHC^, Y504^CHC^ and V508^CHC^ (Fig. 4C). Residue L560^Opt SP TACC3^ occupies a position similar to Y560^WT pTACC3^, but the smaller side chain results in a slight decrease in buried surface area (Fig. 4C–E). A significant change is observed in L566^Opt SP TACC3^, which packs against CHC and occupies a pocket not previously identified in the crystal structure of pTACC3/CHC as defined by CHC residues L452^CHC^, K453^CHC^, E449^CHC^ on the α8-helix, S477^CHC^ and L474^CHC^ on the α10-helix. This appears to be stabilised by the side chain of F567^Opt SP TACC3^, which also packs against the side chain of L560^Opt SP TACC3^ and the methyl group of 561^Opt SP TACC3^. We believe this is not a genuine difference in the position of the side L566 chain but rather an artifact in the model of the WT pTACC3 structure since there was greater ambiguity in the prior electron density map due to lower resolution. Moreover, the new structure provides a clearer structural rationale to explain why L566^TACC3^ is one of the critical hydrophobic residues in the interaction with CHC.

The crystal structure reveals insight into how the stapled peptide overcomes the electrostatic repulsion between the basic residues of WT TACC3 and K507^CHC^. In the Opt SP TACC3 structure, the side chain of E558^Opt SP TACC3^ occupies a similar position to the phosphate group of pS558 of WT pTACC3 in the vicinity of R555^Opt SP TACC3^ and K507^CHC^, potentially forming ionic interactions with either residue (Fig. 4A). E562^Opt SP TACC3^ also reaches close to K507^CHC^, consistent with the improved binding affinity of the stapled peptide due to the substitution of K562 in WT pTACC3. However, it should be noted that the electron density for the K507 side chain is weak, indicating flexibility in this residue. This is consistent with this residue not forming a well-defined salt bridge with Opt SP TACC3.

The stapled TACC3 structure also more clearly shows polar and electrostatic interactions involving D564. This side chain is positioned to form a hydrogen bond with S477^CHC^ and a salt bridge with R481^CHC^. The hydrogen bond between D564^SP TACCC3^ and S477^CHC^ was described in the WT pTACC3/CHC structure (Fig. 4E), however, due to the differences in the conformation of the peptides, the side chain of D564^SP TACC3^ can form a new salt bridge with R481^CHC^. This difference may be genuine, but it may also reflect the improved map in the Opt SP TACC3 structure. In addition to intermolecular hydrogen bonding with CHC, D564^SP TACC3^ also forms intramolecular hydrogen bonds in the crystal structure. The D564^Opt SP TACC3^ side chain connects with the main chain amide of L566^Opt SP TACC3^ (L566_HN_–D564_CγO_ 2.2 Å), and the Asp amide carbonyl can interact with the main chain amide of F567^Opt SP TACC3^ (F567_HN_–D564_CO_ 2.4 Å). Collectively this gives rise to a turn in the TACC3 backbone around P565 that helps to correctly position surrounding residues to allow them to interact with CHC. To further analyse this internal feature within TACC3, NMR spectroscopy was also performed (Fig. S5). The TACC3 peptide appears disordered in CD or NMR measurements, independent of phosphorylation state, albeit with some limited helical propensity prior to the phospho-site (Fig. S2). However, from ^1^H–^15^N HSQC spectra of TACC3 samples recorded at different temperatures, L566 and L567 peaks do not move with increasing temperature and thus have much lower temperature coefficients compared to the rest of TACC3 (Fig. S5A, B). This is an indicator of a degree of protection of the HN group from solvent, typically resulting from hydrogen bonding interactions as would be afforded by D564^66^. A sole long-range NOE between L567_HN_ and one of the D564_Hβ_ protons was observed at low temperature (Fig. S5C, D), and the NOE between the L566_HN_ and L567_HN_ protons persists up to room temperature (Fig. S5E). These protons must remain close enough in space to allow the NOE to develop, thus indicating limited flexibility of the chain in this region. These findings are compatible with the distances observed between equivalent atoms in the Opt SP TACC3–CHC crystal structure (L566_HN_–F567_HN_ 2.6 Å, F567_HN_–D564_Hβ_ 3.7 Å). Thus, it appears that this structural feature exists within unbound TACC3/pTACC3 and is a phosphorylation-independent component of the interaction.

### Stapled TACC3 peptides are efficient cell penetrating molecules

**Fig. 5:**
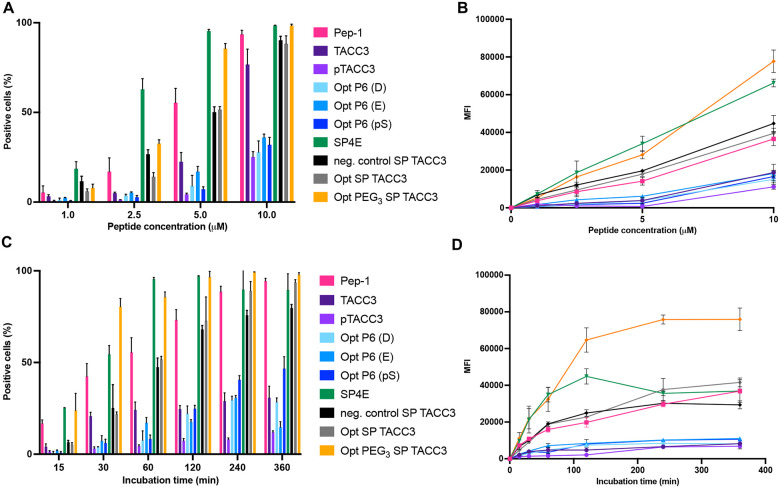
Effects of peptide concentration and incubation time on cellular uptake of FAM-conjugated WT, optimised and stapled TACC3 peptides by HeLa cells, measured by flow cytometry. **A** The ratio of cell containing internalised peptides (%) and **B** mean fluorescence intensity (MFI) measurements obtained by flow cytometry show a dose-dependent increase in cell internalization. **C** Percentage of cells with internalised peptides and **D** mean fluorescence intensity (MFI) measured in HeLa cells over time at 5 μM concentration in the cell culture medium. Error bars represent the SD of at least three independent biological replicates.

The results of cell permeability studies suggest the effect of concentration on the uptake mechanism can be a complex balance of both vesicular and direct penetration, with endocytosis occurring predominantly at low peptide concentrations, and simultaneous vesicular and direct penetration detected at higher concentration^67,68^. The cell permeability of the original TACC3 sequences (TACC3 & pTACC3), sequence optimised peptides (Opt P6 [D/E/pS]) and stapled peptides (Opt SP TACC3 & Opt PEG_3_ SP TACC3) were compared to cell penetration characteristics of known cell penetrator Pep-1 (KETWWETWWTEWSQPKKKRKV)^69–71^. Pep-1 was chosen based on a comparable permeability assessment in live HeLa cells using quantitative flow cytometry where its uptake exceeded other commonly used permeable peptides^62^. HeLa cells were incubated with peptides, thoroughly washed and trypsinised to remove surface-bound peptides. Flow cytometry was used to quantify the percentage of fluorescent-positive cells. Cellular peptide uptake was further assessed using mean fluorescence intensity (MFI) analysis of the flow data. Cellular uptake at increasing peptide concentrations showed a dose-dependent accumulation (Fig. 5A, 5B). The experiments were repeated at a fixed peptide concentration over a time course to determine the kinetics of uptake (Fig. 5C, 5D). The native unphosphorylated TACC3 peptide exhibited low and slow cellular uptake, and the phosphorylated TACC3 peptide was even worse, confirming the predicted effect of the negatively charged phosphate group in retarding uptake. The non-stapled versions of the sequence-optimised peptides also had low cell membrane permeability. In contrast, cell penetration of Pep-1 and stapled TACC3 peptides was significantly higher, even at low micromolar concentrations. Although cellular uptake of non-stapled peptides improved at higher peptide concentration (10 μM) and longer incubation times, respectively, stapled peptides consistently exhibited 5–10-fold higher intracellular fluorescence across all concentrations and time points. Notably, the cellular uptake of the TACC3 stapled peptides was comparable to the model Pep-1 peptide. Thus, the introduction of the staple into the peptide not only improved the binding of a pre-organised peptide to its target but also enabled efficient cellular delivery.

### Stapled TACC3 peptides displace endogenous TACC3 from the mitotic spindle

**Fig. 6:**
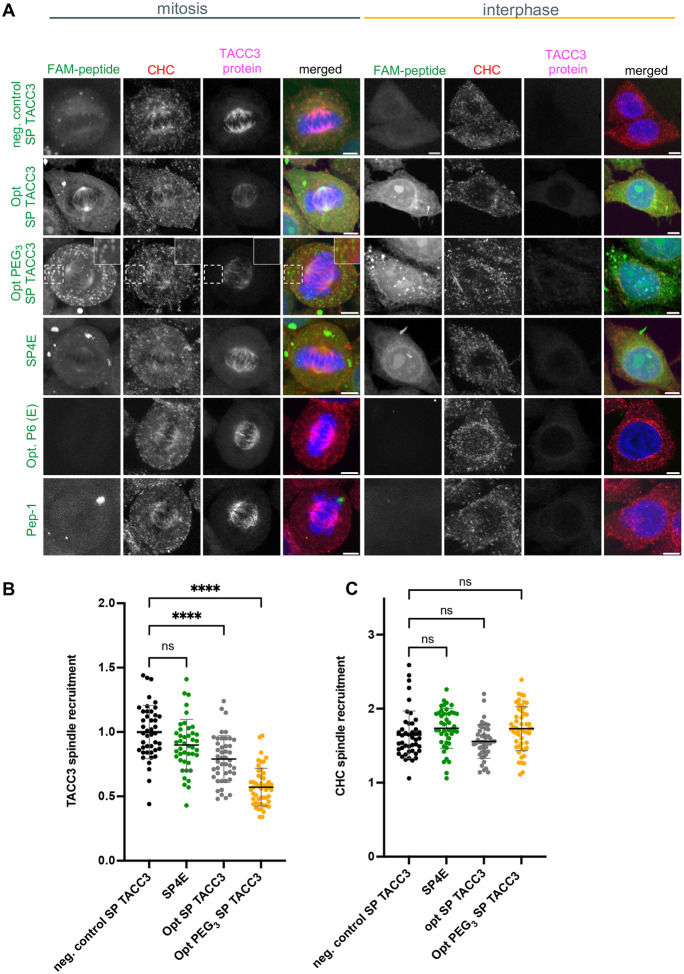
TACC3 stapled peptides localise into the mitotic spindle and displace endogenous TACC3 protein in mitotic HeLa cells. **A** Representative confocal images of fixed HeLa cells during metaphase and interphase 5 h after adding 5 μM FAM-TACC3 peptides (green) to the cell culture media. Unstapled Opt P6 (E558) is not sufficiently cell permeable. Cells were stained for endogenous full length CHC (red), full length TACC3 (purple) and DNA (DAPI, blue). Scale bars: 5 μm. **B** Quantification of endogenous TACC3 localization on mitotic spindles of cells in metaphase in cells treated with 5 μM FAM-TACC3 peptides from the representative images, normalised to the average negative control fluorescence value. Each dot represents a single cell, n=43–51 cells per each peptide measurement pooled from three independent experiments. The short bar and error bars show the mean ± SD. **C** Quantification of the spindle localisation of CHC, expressed as a ration of spindle bound CHC to the cytoplasmic fraction. Each dot represents a single cell, cell count identical to (**B**). ANOVA with Dunnett’s post-hoc test was used to compare the mean of each group to the mean of the control group. The *p*-value is shown compared to the negative control SP TACC3 peptide: **** *p*<0.0001; ns *p*>0.05.

The subcellular localisation of the FAM-labelled stapled peptides was examined by confocal microscopy in fixed HeLa cells (Fig. 6). The stapled peptides were highly cell permeable within the cells, with a diffuse fluorescent signal observed in the cytoplasm, nuclei and nucleoli of interphase cells. Despite the potential for fluorescence quenching due to the cell fixation process^72^, the stapled peptides remained clearly visible without the need for additional antibody signal enhancement. During mitosis, the three different stapled TACC3 peptides (Opt. SP TACC3, Opt PEG3 SP TACC3, SP4E), colocalised with endogenous TACC3 on the mitotic spindle, but not the non-CHC-binding stapled TACC3 peptide (neg. control SP TACC3) (Fig. 6A). The Opt PEG_3_ SP TACC3 peptide also localised to distinct puncta. This signal did not colocalise with the clathrin-coated vesicles in the cytoplasm, and we conclude it may arise from aggregation or concentration-dependent liquid–liquid phase separation, which has been previously observed for pegylated proteins and peptides within the cellular environment^73^.

The capability of the peptides to displace endogenous TACC3 protein from mitotic spindles was quantified in metaphase where HeLa cells were treated with increasing peptide concentration (2.5, 5 and 10 μM). Highly cell permeable peptides were observed to localise to the mitotic spindle, however only the sequence-optimised stapled peptides significantly reduced the binding of full-length endogenous TACC3 protein to the microtubules. The levels of endogenous TACC3 in the mitotic spindle were reduced by ~20% with Opt SP TACC3 and up to 40% with Opt PEG_3_ SP TACC3 compared to the negative control SP TACC3 peptide (Fig. 6B, S6). This indicates that the Opt SP TACC3 peptides effectively disrupt the CHC/TACC3 interaction, resulting in reduced TACC3 localisation to the mitotic spindle of HeLa cells. In contrast, stapled peptides did not affect the recruitment of CHC to the spindle from the cytoplasm (Fig. 6C). This is consistent with previous studies using genetic approaches to disrupt TACC3, in which a slight reduction in clathrin localisation to the spindle was observed only after the complete removal or relocalisation of TACC3 from the spindles of mitotic cells^30,33^. Due to concerns about the potential for non-specific effects caused by the aggregation of Opt PEG_3_ SP TACC3, we focused on using Opt SP TACC3 as the disruptor for further functional studies.

### CHC/TACC3 interaction inhibitors delay mitotic progression

**Fig. 7:**
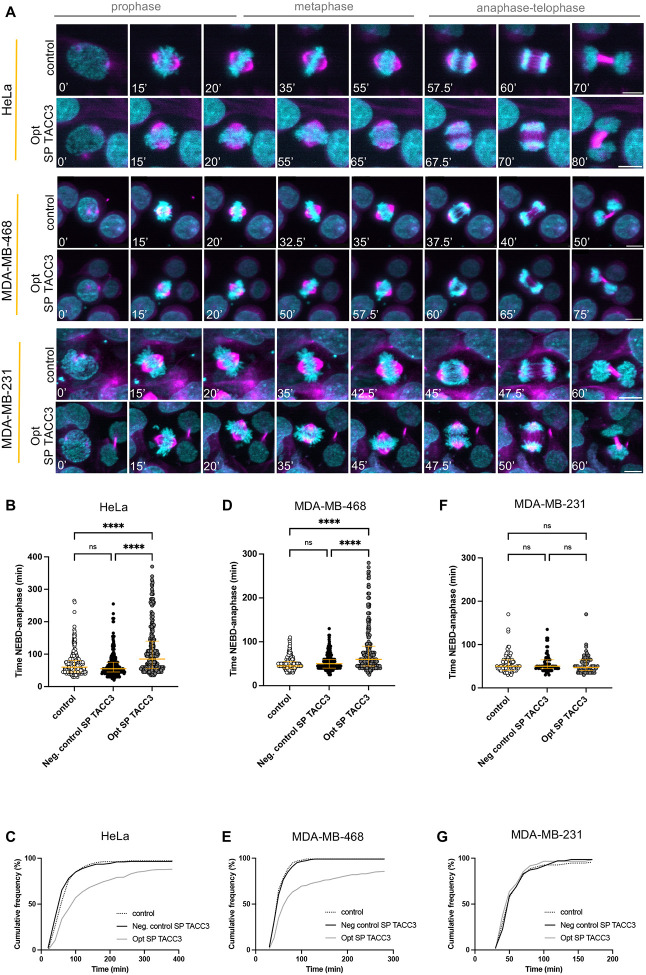
Treatment of cells with stapled peptides results in a slower progression through mitosis. **A** Representative images of control and peptide treated HeLa, MDA-MB-468 and MDA-MB-231 cells progressing through mitosis, stained with SiR-tubulin (magenta) and SPY555-DNA (cyan). Numbers in white indicate the time from NEBD (nuclear envelope breakdown) to telophase of the cells. Scale bars: 10 μm. Data were collected for 5 h, with data acquisition every 2.5 min; **B, C** Scatter plots and cumulative histograms of the time in mitosis from NEBD to anaphase onset in SiR-tubulin labelled HeLa cells treated with 5 μM neg. control SP TACC3, Opt SP TACC3 and a control treated with DMSO carrier; each dot represents a single cell (246–297 mitotic events over 3 imaging sessions) Data were collected for 10 h, with data acquisition every 5 min; **D, E** Scatter plots and cumulative histograms of the time in mitosis of MDA-MB-468 cells (230–261 mitotic events over 3 imaging sessions) and **F, G** MDA-MB-231 cells (59–93 mitotic events over 3 imaging sessions) were imaged under identical conditions. The plots indicate the median and interquartile ranges (25th–75th percentile). Kruskal-Wallis test results show the *p*-value: **** *p*<0.0001, ns *p*>0.05.

The impact of disrupting the CHC/pTACC3 interaction was assessed using time-lapse microscopy of live HeLa cells stained with SiR-tubulin. The median time of mitosis from nuclear envelope breakdown (NEBD) to anaphase onset in control HeLa cells was 55–60 min (Fig. 7A, B). Treatment of HeLa cells with 5 μM Opt SP TACC3 led to a prolonged median duration of mitosis to 85 min from NEBD to the anaphase onset, compared to 60 min for the control cells and 55 min for negative control SP TACC3 treated cells (Fig. 7A, B, C). Thus, a small but significant increase was observed in the duration of prophase, while the primary effect was the extension of metaphase duration (Fig. S8). The proportion of HeLa cells exiting metaphase during the course of cell imaging decreased from 97% to 88% when treated with Opt SP TACC3 (Fig. 7C).

TACC3 is frequently overexpressed in breast cancer^74^ and was shown to be particularly important for the survival of cells exhibiting centrosome amplification^38,75^. However, the functional significance of the CHC/TACC3 interaction is unknown in this context. To probe this, we selected two triple-negative breast cancer (TNBC) cell lines (MDA-MB-468 and MDA-MB-231) in which to apply the TACC3 stapled peptides.

Peptide uptake was measured in both cell lines, confirming that the stapled peptides were permeable (Fig. S7). The median time of mitosis for control cells of both TNBC cell lines was ~50 min and 95–99% of cells exited metaphase. However, MDA-MB-468 showed a delay in progression from NEBD to anaphase onset of ~10 min after treatment with 5 μM SP TACC3, an effect limited entirely to the metaphase stage (Fig. 7A, D, E, S8), with 85% of cells reaching anaphase during the imaging experiment. On the other hand, mitosis in MDA-MB-231 cells appeared unaffected by the peptide (Fig. 7A, F, G, S8). The peptide did not affect the viability of HeLa, MDA-MB-231 or MDA-MB-468 cells (Fig. S9).

## Discussion

TACC3 and CHC are core components of a multiprotein complex that binds spindle microtubules and stabilises k-fibres of the mitotic spindle^26,30^. The regulation of the interaction between TACC3 and CHC by the mitotic kinase Aurora-A is essential for the proper and timely formation of the mitotic spindle. Disruption of the TACC3 docking motif for binding on Aurora-A, which prevents TACC3 phosphorylation, impaired the localization of TACC3 on the mitotic spindle and delayed the completion of mitosis^26,31^. Here we provide mechanistic insights into the interaction of phosphorylated TACC3 with CHC, and developed a peptidomimetic inhibitor that confirms the contribution of the interaction to mitotic timing.

TACC3 is a member of the centrosomal TACC family characterised by their conserved ~200 amino acid C-terminal coiled-coil domain (TACC domain)^22,76^. Phylogenetic studies suggest that both *TACC3* and *AURKA* evolved from single ancestors via subsequent successive duplications of the ancestral genome during vertebrate evolution^76–78^. Therefore, invertebrates have a single known TACC gene (Spc72 in *S. cerevisiae*, TACC in *D. discoideum*, TAC1 in *C. elegans*, and D-TACC in *D. melanogaster*)^22,79–81^. A single TACC gene was identified in the genome of the tunicate *Ciona intestinalis*, while all vertebrates contain three TACC protein isoforms TACC1, TACC2, and TACC3^76^. *X. laevis* was considered an exception with just one isoform TACC3/Maskin initially identified^82^, however, it was recently reported that *Xenopus*, like all other vertebrates, contains three TACC family members^83^. Sequence alignment of TACC3 protein sequences shows not only an evolutionary conserved TACC domain, but also a high level of conservation in the Aurora-A and clathrin interacting domains in TACC3 orthologues despite having considerable variability in both size and sequence. Reaching upstream from S558 up to F525^TACC3^, the key interaction residues are conserved in TACC3 as well as in Aurora-A orthologs. A high level of sequence identity has also been reported for the sequence essential for the interaction with clathrin, involving the hydrophobic residues together with S558 and a positively charged side chain at position S+4^26^. Clathrin sequences characterised by a high sequence conservation among taxons. The phylogenetically conserved sequence alignment of TACC3 and clathrin can be extended up to evolutionarily primitive organisms such as phylum Cnidaria, pointing to a potentially ancient and conserved structural mechanism of regulation of mitotic spindle dynamics in metazoans (Fig. S10).

Interactions of phosphorylated proteins typically involve a canonical phosphoreader domain such as SH2^84^ or BCRT^85^, which bind their targets in an extended conformation and recognise the phosphate group directly often using multiple basic residues to achieve high affinity binding. CHC does not have a canonical phosphoreader domain, and its interaction with phosphorylated TACC3 follows a different set of rules. The first rule is there is a significant phospho-independent component, involving helices 8 and 10 of CHC and the “DPLL” motif of TACC3, which forms a stable structure in solution. The second rule is that CHC recognises a helical conformation of TACC3. Phosphorylated pS558 can interact intramolecularly with the positively charged R*(i* − 3) and K(*i* + 4), a context in which phosphoserine has a stabilising effect on α-helix formation in model peptides^49^. However, in TACC3, the effect is a subtle alteration of the helical propensity along the sequence, with no clear overall helical stabilisation. Notably however, the helical context of pS558 is important for the display of hydrophobic residues that drive the interaction with CHC. The third rule of this interaction is the single basic residue of CHC in the interface, K507, does not contribute much to binding affinity, but is nonetheless crucial for selection of the phosphorylated form of TACC3 versus non-phosphorylated TACC3. In this unusual mechanism, the phosphorylation of S558 alters the charge distribution at the interaction interface and mitigates the repulsion between the positively charged surfaces of the WT protein. This is important for fine-tuning the phosphor-dependent selectivity of the interaction which is otherwise largely driven by hydrophobic interactions.

The CHC/TACC3 interaction now joins a select group of systems which do not involve typical phosphoreader domains that have been studied at the molecular level and have contributed to the development of PPI inhibitors. Skinner at al.^6^ identified a salt bridge competition mechanism that enables a phospho-triggered swap of protein partners by Raf Kinase Inhibitory Protein (RKIP). Upon phosphorylation, RKIP transitions from inhibiting Raf-1 to inhibiting G-protein-coupled receptor kinase 2. In this interaction a phosphoserine disrupts an existing salt bridge, initiating a partial unfolding event and promoting new protein interactions^6^. CREB-binding protein (CBP) is a transcriptional coactivator in eukaryotic transcriptional regulatory networks, interacting with multiple disordered and partially ordered partners. The interaction of the KIX domain of CBP with transcription factors involved in hematopoietic differentiation (CREB, c-Myb, MLL) has been studied in detail and provides examples of phosphorylation, induced fit and conformational selection at the same binding site^86,87^. The KID domain of the inducible transcriptional activator CREB must be phosphorylated on S133 to bind to the interaction interface of KIX with high affinity. This phosphoserine does not form a salt bridge with side chains in its proximity; instead it forms an intermolecular hydrogen bond with T658 of KIX and intramolecular hydrogen bond with R131 of pKID^88–90^. Detailed information on these PPIs has enabled structure-guided molecular design of a peptidomimetic inhibitor targeting the cMyb-KIX interaction with convincing pro-apoptotic anti-leukemic effect^91,92^.

Depletion of TACC3 in HeLa cells induces phenotypes characterised by chromosome misalignment accompanied by a mitotic delay^22,26,93^; aberrant spindles have also been observed^23^. The mechanism may be linked to the inactivation of p53 in HeLa cells^94^, which is associated with an impaired postmitotic checkpoint. Consequently, TACC3 depletion results in prolonged activation of the spindle assembly checkpoint, arresting cells in metaphase until all chromosomes are properly attached^95^. The stapled inhibitory peptides targeting the PPI of pTACC3 with CHC in HeLa cells demonstrated high target specificity, resulting in prolonged mitotic timing despite only partial removal of native TACC3 from the spindles, without measurable toxic effects on other cellular processes. The phenotype induced by the stapled peptide (median NEBD-to-anaphase timing of 85 min) is comparable to the effect of relocalising the endogenous CHC/TACC3 complex using a rapalog-sensitive MitoTrap (median NEBD-to-anaphase timing of 73.5 min)^30^. Only one of the two breast cancer cell lines tested, MDA-MB-468, also showed a mitotic delay phenotype. This cell line was selected on the basis that it is highly dependent on both TACC3 and clathrin gene expression; based on analysis by Project DRIVE^96^ MDA-MB-468 has a gene expression sensitivity score of −1.202 for TACC3 and −1.988 for CLTH (clathrin). By the same criteria, MDA-MB-231 is not dependent on TACC3 (TACC3 expression sensitivity score of 0.612), but rather is characterised by a high TACC3 expression associated with centrosome amplification. These cells require an elevated level of TACC3 protein during mitosis due to its function in centrosome clustering as for successful cell division^38^￼. Higher TACC3 expression in these cells may protect them from delays in mitosis upon treatment with the Opt SP TACC3 disruptor. Previous studies have also reported differing responses of the two triple negative breast cancer cell lines to TACC3 inhibitors, which may reflect distinct functional interactions of TACC3 throughout^38,97^￼. Tools that disrupt the protein–protein interactions such as Opt SP TACC3 and Affimer-E8 (a disruptor of the TACC3/ch-TOG interaction^98^￼ enable greater precision in dissecting the functions and cellular dependencies of TACC3.

Peptide therapeutics are often characterised by suboptimal pharmacokinetics, including fast serum degradation and poor circulating half-lives. As the α-helix represents a typical recognition motif at PPI interfaces, stapled peptides have been engineered to target inhibition of PPIs involving helix-adopting protein sequences with high affinity, with the added benefits of improved membrane permeability and resistance to proteolytic degradation in biological systems^43–45,60–62,99^. Several computational methods and high-throughput analyses have been developed to investigate the cell-penetration properties of stapled peptides to speed up the development of therapeutic peptides. These analyses focus largely on the peptide sequence, charge and staple positions. Systematic analyses of peptide libraries have been employed to evaluate the effects of hydrophobicity and peptide charge on peptide uptake^43,100,101^. In this work, the development of a stapled peptide inhibitor started with the use of peptide arrays, complemented with molecular modelling, to choose sequence modifications to enhance peptide binding without the need for phosphorylation. Sequence optimisation resulted in a ~150-fold increase in the binding affinity of the phosphomimetic peptide in comparison to the WT sequence. The incorporation of a site-specific staple into this TACC3 peptide further improved binding affinity with CHC by pre-organising and constraining the peptide into an α-helical conformation. Structural analysis confirmed the staple is positioned on the solvent-exposed side of the peptide that does not disrupt PPI interface. The combination of sequence optimisation and stapling enabled the peptide to bind the CHC interface with high affinity without requiring phosphorylation, which would negatively affect cell internalisation. The increased affinity of the stapled peptide facilitated the collection of much higher-resolution crystal structure data than had previously been achieved for the TACC3/CHC interface. Structural analysis allowed us to infer in greater depth the mode of binding of WT pTACC3 to CHC in terms of the positioning of the TACC3 hydrophobic residues as well as the beneficial charge balancing of basic residues around the phosphoryl group. Beyond its high affinity for the target, the stapled phosphomimetic peptide exhibits very high cellular uptake and cytosolic distribution, surpassing the best linear cell-penetrating peptides like Pep-1^43,62^. In summary, this work highlights the value of developing staple peptide inhibitors as research tools/probes for enabling more precise structural and cellular biology studies of the target interaction.

## Supplementary Material

Supplementary Files

This is a list of supplementary files associated with this preprint. Click to download.

- Manuscripttableandfigures.pdf
